# Potential for a Plant-Made SARS-CoV-2 Neutralizing Monoclonal Antibody as a Synergetic Cocktail Component

**DOI:** 10.3390/vaccines10050772

**Published:** 2022-05-12

**Authors:** Collin Jugler, Haiyan Sun, Francisca Grill, Karen Kibler, Adrian Esqueda, Huafang Lai, Yize Li, Douglas Lake, Qiang Chen

**Affiliations:** 1 The Biodesign Institute, Arizona State University, Tempe, AZ 85287, USA; collin.jugler@asu.edu (C.J.); haiyan.sun@asu.edu (H.S.); karen.kibler@asu.edu (K.K.); adrian.esqueda@asu.edu (A.E.); huafang.lai@asu.edu (H.L.); yize.li.2@asu.edu (Y.L.); douglas.lake@asu.edu (D.L.); 2 School of Life Sciences, Arizona State University, Tempe, AZ 85287, USA; fgrill@asu.edu

**Keywords:** SARS-CoV-2, COVID-19, monoclonal antibody (mAb), plant-made antibody, antibody cocktail, neutralization synergy, plant-made pharmaceutical

## Abstract

Severe acute respiratory syndrome coronavirus 2 (SARS-CoV-2) has caused a public health crisis over the last two years. Monoclonal antibody (mAb)-based therapeutics against the spike (S) protein have been shown to be effective treatments for SARS-CoV-2 infection, especially the original viral strain. However, the current mAbs produced in mammalian cells are expensive and might be unaffordable for many. Furthermore, the emergence of variants of concern demands the development of strategies to prevent mutant escape from mAb treatment. Using a cocktail of mAbs that bind to complementary neutralizing epitopes is one such strategy. In this study, we use *Nicotiana benthamiana* plants in an effort to expedite the development of efficacious and affordable antibody cocktails against SARS-CoV-2. We show that two mAbs can be highly expressed in plants and are correctly assembled into IgG molecules. Moreover, they retain target epitope recognition and, more importantly, neutralize multiple SARS-CoV-2 variants. We also show that one plant-made mAb has neutralizing synergy with other mAbs that we developed in hybridomas. This is the first report of a plant-made mAb to be assessed as a potential component of a SARS-CoV-2 neutralizing cocktail. This work may offer a strategy for using plants to quickly develop mAb cocktail-based therapeutics against emerging viral diseases with high efficacy and low costs.

## 1. Introduction

The global pandemic caused by severe acute respiratory syndrome coronavirus 2 (SARS-CoV-2), has caused an unprecedented public health crisis. SARS-CoV-2 is a betacoronavirus of the family *Coronaviradae*, housing a large, positive-sense, single-stranded RNA genome [1]. The disease caused by SARS-CoV-2 is known as coronavirus disease 2019 (COVID-19) and the severity of symptoms observed in patients infected with SARS-CoV-2 vary widely [2]. Although several vaccines are available that are effective at preventing severe disease [3,4], the persistence of SARS-CoV-2 infections and the emergence of variants of concern urges the continual development of prophylactics and therapeutics that can be used to treat infected individuals or prevent infections in people who do not meet vaccine eligibility.

The SARS-CoV-2 viral particle utilizes the spike (S) glycoprotein for binding to the cellular receptor angiotensin-converting enzyme 2 (ACE2), mediating entry into cells [5,6]. The S trimer protrusions on the viral surface are composed of three S1 subunits, containing the receptor-binding domain (RBD) and N-terminal domain (NTD), as well as three S2 subunits that include the fusion peptide necessary for membrane fusion [7]. Structural analyses show that the three RBDs on the trimer show a flexible nature, where RBDs may be in either an “up and open”, a “down and closed”, or an intermediate conformation where the RBD is closed, yet shows more mobility than the “down and closed” position [8,9]. The receptor accessible conformation of the RBD is the “up and open” form, and, upon the interaction of at least one RBD with ACE2, the S trimer undergoes a structural transition, ultimately leading to the fusion of S2 with the host membrane, promoting viral entry [10].

Monoclonal antibodies (mAbs) are a rapidly expanding class of biological therapeutics used to treat a host of conditions, from cancer to infectious diseases [11]. Several groups have identified neutralizing monoclonal antibodies (nAbs) against SARS-CoV-2 from B cells of convalescent individuals or humanized mice [12,13,14,15,16], and some larger entities have completed or advanced nAbs or cocktails of nAbs in clinical trials [17], validating the utility of nAb countermeasures against SARS-CoV-2. The available structural data on SARS-CoV-2 S protein functional domains and conformations, as well as the inherent antigenicity of the S glycoprotein, assist in the development and characterization of nAbs. The mechanism by which many of the nAbs inhibit SARS-CoV-2 infection is by binding to the S protein and interfering with binding to the ACE2 receptor at the RBD; however, nAbs have been identified whose epitope is at the N-terminal domain as well [16,18]. Furthermore, mAbs against the RBD can be categorized into four classes based on their RBD epitope and the RBD conformation [19,20]. Classes 1 and 2 contain mAbs, whose epitope overlaps with the ACE2 binding site and are generally effective neutralizers. Class 3 mAbs do not directly overlap with the ACE2 binding site, yet they still potently neutralize the virus. Class 4 mAbs do not inhibit ACE2 binding and are generally less potent in neutralizing capacity. Such a classification system is useful for characterizing nAbs for use in antibody cocktails. The development of antibody cocktails composed of nAbs that have non-redundant epitopes reduces the risk of viral escape due to mutations in the RBD, the dominant target for nAbs [21]. The ongoing need for developing new nAb cocktails is highlighted by the emergence of variants of concern that have mutations in previously identified neutralizing epitopes, thereby reducing the efficacy of previously potent nAbs [20,22,23]. Specifically, several nAbs that received emergency use authorization (EUA) by the Food and Drug Administration (FDA) for the treatment or prevention of COVID-19, have been revoked due to the emergence of the dominant omicron variant and the reduced activity these mAbs have against this variant [24,25]. A rational design of cocktails by characterizing nAbs without overlapping epitopes that are essential targets on the S protein, will be required for efficacious antibody cocktail therapies as SARS-CoV-2 continues to evolve and circulate globally.

Plants are a versatile and alternative system that is useful for producing mAbs [26]. As eukaryotic organisms, they are capable of post-translational modifications, such as glycosylation, which is essential to the functionality of many pharmaceutical proteins, specifically mAbs [27,28,29,30]. Plants also offer other advantages over traditional methods of pharmaceutical protein production, such as lower costs, increased safety, and a simple scale-up ability for commercial manufacturing [31]. The potential of plants to contribute to biologics development against SARS-CoV-2 has been highlighted by generating diverse mAbs and several vaccine candidates, with one plant-made vaccine currently approved in Canada [32,33,34,35,36,37,38,39,40]. Here, we contribute further to the development of nAbs that may be useful as combinations in cocktails. Two nAbs are expressed in *Nicotiana benthamiana*, biochemically characterized, and are analyzed for their potential efficacy as combinatorial therapies. We highlight the need to continue the development of effective and economically feasible nAbs for use as cocktail components against SARS-CoV-2 as new variants emerge.

## 2. Methods and Materials

### 2.1. Plant Expression of CA1 and CB6

The variable heavy (VH) and variable light (VL) gene sequences for CA1 and CB6 [15] were codon-adapted for plant-based expression using GeneDesigner 2.0 and synthesized by Integrated DNA Technologies (IDT). The VH and VL sequences were ligated to human constant regions (kappa for the light chain and gamma for the heavy chain), cloned into a geminivirus-based plant expression vector, and transformed into *Agrobacterium tumefaciens* for agroinfiltration, as described previously [41]. Six-week-old *Nicotiana benthamiana* plants were agroinfiltrated and leaves were harvested at peak recombinant protein expression (7 days post infiltration).

### 2.2. mAb Extraction and Purification

MAbs were extracted from *N. benthamiana* leaves and subjected to Protein A affinity chromatography, as previously described [41,42,43]. Briefly, plant leaves expressing a mAb were blended in a 1:1.5 ratio of fresh leaf mass: extraction buffer. Extraction buffer consisted of 1× phosphate-buffered saline (PBS), pH 5.2, 10 mg/mL of sodium L-ascorbate, 1 mM ethylenediaminetetraacetic acid (EDTA), and 2 mM phenylmethylsulfonyl fluoride (PMSF). The plant extract was clarified by centrifugation and host protein precipitation overnight at pH 5.2. The clarified extract was filtered through a 0.22 µm filter prior to enrichment and purification using MabSelect (GE Healthcare, now Cytiva, Chicago, IL, USA) resin for affinity chromatography.

### 2.3. SDS-PAGE and Western Blotting

Purified, plant-made CA1 and CB6 were assessed by SDS-PAGE and Western blot analysis, as described [41,44]. Briefly, purified CA1 and CB6 were subjected to SDS-PAGE under reducing and non-reducing conditions and separated on 4–20% gradient polyacrylamide gels (Bio-Rad). The gels were then stained with Coomassie Brilliant Blue R-250 or proteins were transferred to PVDF membranes. The membranes were then probed with either goat anti-human kappa chain (Southern Biotech, Birmingham, AL, USA, Cat. No. 2060-05) or goat anti-human IgG (Southern Biotech, Cat. No. 2040-05) conjugated to horseradish peroxidase (HRP), prior to development with the Pierce ECL Western blotting substrate (Thermo Scientific, Waltham, MA, USA) and image capture with an ImageQuant instrument.

### 2.4. Temporal Expression ELISA

Temporal analysis of mAb expression in plant leaves was performed, as described previously [30]. Briefly, goat anti-human IgG (Southern Biotech, Cat. No. 2040-01) was coated in a carbonate-bicarbonate buffer on high-binding, 96-well plates at a concentration of 2 µg/mL at 4 °C overnight. Wash steps were performed with phosphate-buffered saline with 0.05% Tween^®^20, pH 7.4 (PBST), four times between each step. Leaves harvested at one-day intervals after agroinfiltration were blended as described above and a two-fold dilution series was added to the plate, alongside a human IgG standard curve dilution series. Goat anti-human kappa chain conjugated to HRP (Southern Biotech, Cat. No. 2060-05) was used to detect the captured plant-made mAbs. A KPL 3,3′,5,5′-tetramethylbenzidine (TMB) substrate (SeraCare Life Sciences Inc., Milford, MA, USA) was used in conjunction with a 1M H_2_SO_4_ stop solution. Absorbance data were analyzed with GraphPad Prism to calculate the microgram of recombinant antibody per gram of fresh leaf weight.

### 2.5. RBD-Binding ELISA

Recombinant RBD from the WA1/2020 strain was coated in a carbonate-bicarbonate buffer on high-binding 96-well plates at a concentration of 2 µg/mL at 4 °C overnight. Wash steps were performed with 1× PBST, four times between each step. Purified, plant-made CA1 or CB6 dilutions were added to the plate, and antibodies bound to RBD were detected with goat anti-human IgG conjugated to HRP (Southern Biotech, Cat. No. 2040-05). A KPL TMB substrate was used in conjunction with a 1M H_2_SO_4_ stop solution to develop the plates. Absorbance data were analyzed with GraphPad Prism software with a *K_D_* value calculated by nonlinear regression analysis using a one-site binding model.

### 2.6. Competitive Sandwich ELISAs

Serial dilutions of each antibody (plant-made CA1 and CB6, and hybridoma-made 3C4 and 11D7) were coated overnight at 4 °C in 96-well plates. The plates were then incubated with recombinant WA1/2020 RBD at 2 µg/mL for 1 h at 37 °C. After washing with 1× PBST, the plates were further incubated with either CR3022 or CB6, both conjugated to HRP with the use of the EZ-Link Plus Activated Peroxidase Kit (Thermo Scientific). The plates were then developed with a KPL TMB substrate in conjunction with a 1M H_2_SO_4_ stop solution. Absorbance data were plotted with GraphPad Prism using nonlinear regression.

### 2.7. Viruses and Cells

SARS-CoV-2 (USA-WA1/2020 strain, NR-52281) and delta (NR-55611) were obtained from BEI. The genomic RNA was sequenced and was found to be identical to GenBank: MN985325.1 and OL442162.1. The USA-WA1/2020 strain was propagated in African green monkey kidney cells (E6) (obtained from ATCC), which were cultured in Eagle’s Minimum Essential Medium (EMEM; ATCC catalog #30-2003), supplemented with 2% fetal bovine serum (FBS) and 100 µg/mL of streptomycin/penicillin. The delta strain was propagated in Vero cells (E6) or (CCL81) (obtained from ATCC), which were cultured in Dulbecco’s Modified Eagle’s Medium (DMEM; Gibco catalog No. 11965), supplemented with 10% fetal bovine serum (FBS), 100 U/mL of penicillin, 100 μg/mL of streptomycin, 50 μg/mL of gentamicin, 1mM sodium pyruvate, and 10mM HEPES.

Mouse-adapted SARS-CoV-2 MA10 [45] was obtained from Dr. Ralph Baric’s laboratory (the University of North Carolina at Chapel Hill) and was propagated in Vero TMPRSS2 cells [6] (a gift of Dr. Stefan Pӧhlmann), grown in identical conditions as Vero E6 cells.

### 2.8. Focus-Forming Assay

The neutralization of mAbs against SARS-CoV-2 was performed using a focus-forming assay (FFA), as described [46]. Briefly, Vero E6 cells were plated at 25,000 cells/well in 100 µL of DMEM + 10% FBS in a 96-well clear, flat-bottom tissue culture plate the day before the assay. On the day of assay, antibodies were serial diluted (1:4 dilution) with DMEM + 2% FBS in a 96-well round-bottom plate. SARS-CoV-2 stocks were diluted with EMEM + 2% FBS to 2000 plaque-forming units (PFU) per well, then added to the antibody dilution plate and incubated at 37 °C for one hour before adding to the Vero E6 cells. The cells were incubated at 37 °C for one hour and then a 100 µL/well MEM: methylcellulose overlay was added. The infected Vero E6 cells were further incubated at 37 °C for 24 h, except for the delta variant, where infected cells were incubated for 40 h. The MEM: methylcellulose overlay was removed, and cells were fixed with 4% paraformaldehyde and washed with 0.1% saponin and 0.1% bovine serum albumin (BSA) in 1× PBS, six times. The cells were then stained with plant-produced CR3022 at 2 µg/mL and with goat anti-human IgG-HRP (Sigma, Saint Louis, MI, USA, Cat. No. A0170). Finally, KPL TrueBlue substrate (Seracare Life Sciences Inc.) was added, and the plate was imaged with an AID Spot Reader. The data were analyzed using GraphPad Prism 9 and the neutralization percentage was calculated as follows: (average number of foci in virus only wells—number of foci in antibody-treated well)/average number of foci in virus only wells. Each antibody was tested in triplicate and at least two independent experiments were performed.

### 2.9. Neutralization Synergy Analysis

MAbs were diluted to concentrations corresponding to their IC_20_, IC_25_, and IC_50_. FFA experiments were then performed with diluted mAbs either alone, in dual mAb combinations, or as a triple mAb cocktail. Neutralization data for each individual mAb obtained from these FFAs were then imported into SynergyFinder [47] and analyzed with the HSA [48] and Loewe [49] models to calculate the “predicted” neutralization percentage of the cocktail at IC_20_, IC_25_, and IC_50_, assuming there is no synergistic interaction between the different mAbs in the cocktail. The predicted percent neutralization of the cocktail was then compared with the empirically determined percent neutralization for each mAb combination. Synergy could then be inferred from the observations of increased percent neutralization of the empirically determined IC_20_, IC_25_, and IC_50_, relative to the predicted IC_20_, IC_25_, and IC_50_ from each model.

### 2.10. Generation of Monoclonal Antibodies against RBD

Two BALB/c mice were immunized subcutaneously (SQ) with 15 µg RBD (NR-52309 obtained from BEI) protein mixed with an equal volume of Enhanced Magic Mouse Adjuvant (Creative Diagnostics). Immunization was repeated four weeks after the prime immunization and antibody titers in sera were measured the following week by indirect ELISA. A final immunization of 20 µg RBD in 1× PBS was administered SQ two weeks after the boost injection and mice were sacrificed three days later. Spleens were excised and processed into single-cell suspensions, followed by lysis of erythrocytes with 1× red blood cell lysis buffer (Invitrogen). The splenocytes from the mouse with the highest antibody titer to RBD were used for hybridoma generation using a standard method [50]. Briefly, splenocytes were fused with P3X63Ag8.653 murine myeloma cells using 1 mL of 50% polyethylene glycol solution (Sigma-Aldrich). Fused cells were resuspended in a culture medium containing a hypoxanthine-aminopterin-thymidine selection supplement (Sigma-Aldrich). Cells were plated at 6.75 × 10^4^ splenocytes per well in 96-well tissue culture plates and incubated at 37 °C in a 5% CO_2_ incubator. After ten days, culture supernatants were tested for antibodies to RBD by indirect ELISA. Ten ELISA-positive hybridoma parent wells were selected and subcloned by limiting the dilution of 1 cell per well, then rescreened using the same method. Positive subclones were expanded, and the supernatant was harvested for antibody purification by protein A/G (Thermo Scientific) chromatography. Multiple mAbs to RBD were identified, including two named 3C4 and 11D7. All animal experiments were performed under an Institutional Animal Care and Use Committee–approved protocol at Arizona State University (IACUC protocol #22-1881T).

### 2.11. Indirect ELISA for Hybridoma Screening

Indirect ELISAs were performed using RBD and RBD labeled with biotin (RBD-B) using EZ-Link NHS-LC Biotin reagent (Thermo Scientific). For RBD, the antigen was coated on an ELISA plate (Corning) at 1 µg/mL overnight at 4 °C, washed three times with 1× PBST, then blocked in 1% BSA in 1× PBS. For RBD-B, an ELISA plate was coated with 10 µg/mL of streptavidin (Jackson ImmunoResearch, West Grove, PA, USA) for 1 h at 37 °C, and washed three times with 1× PBST, blocked in 1% BSA in PBS, followed by the addition of rRBD-B at 0.5 µg/mL. The plates with RBD-B were washed three times prior to the addition of the primary antibody. Various mouse serum dilutions in 1% BSA in 1× PBS, or neat hybridoma culture supernatants, were added to the wells and allowed to incubate for 1 h. Plates were subsequently washed three times with 1× PBST and then incubated with goat anti-mouse IgG Fc horseradish peroxidase-conjugated antibody (Jackson ImmunoResearch, Cat. No. 115-035-071) for 1 h. Plates were washed four times with 1× PBST and then incubated with TMB substrate (BD Biosciences) for ten minutes. Sulfuric acid 0.16 M was used to stop the reaction and the absorbance at 450 nm was measured using a microplate reader.

## 3. Results

### 3.1. Neutralizing Monoclonal Antibody (nAb) Expression in Nicotiana benthamiana

**Figure 1:**
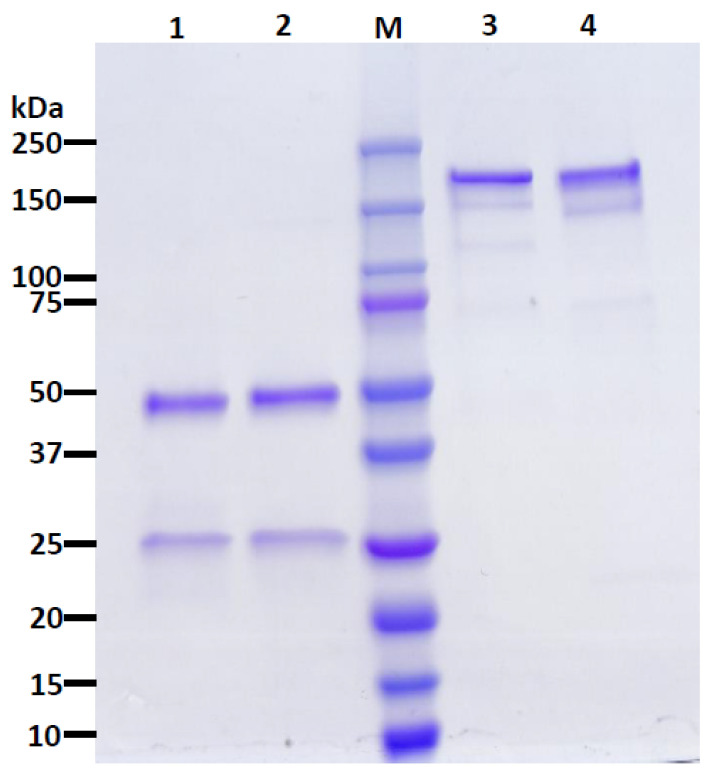
**Purification of CA1 from *Nicotiana benthamiana* leaves.** CA1 purified by Protein A affinity chromatography was subjected to SDS-PAGE under reducing (Lane 1) or non-reducing (Lane 3) conditions on a 4–20% gradient polyacrylamide gel, and the total protein content was stained with Coomassie Brilliant Blue. Approximately 2.5 µg of IgG was loaded in each lane. Lanes 1 and 3, plant-made CA1; Lanes 2 and 4, a mammalian cell-produced anti-West Nile virus E protein (E16) IgG control; M, molecular weight ladder. One representative gel of several experiments is shown.

**Figure 2:**
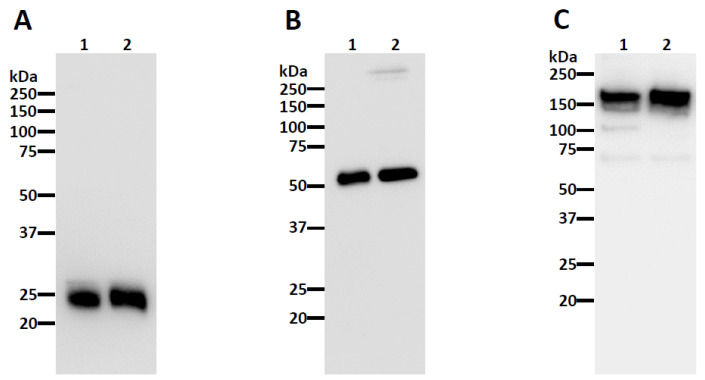
**Western blot analysis of plant-made CA1.** Plant-made CA1 was subjected to SDS-PAGE under reducing conditions (**A**,**B**) and non-reducing conditions (**C**). Proteins were transferred to a PVDF membrane after separation and a horseradish peroxidase-conjugated goat anti-human kappa (**A**,**C**) or goat anti-human IgG (**B**) antibody was used to detect the light chain and heavy chain, respectively. Lane 1, plant-made CA1; Lane 2, mammalian cell-produced anti-West Nile virus E protein (E16) IgG. The blots are representatives of multiple independent experiments.

The nAbs CA1 and CB6 [15] were chosen for expression in *N. benthamiana*. After agroinfiltration [51,52] of the plant codon-adapted gene constructs, the recombinant IgGs were purified by Protein A affinity chromatography. The analysis of the purified nAbs by SDS-PAGE shows that the nAbs were purified to high homogeneity, resulting in a similar purity to a mammalian-produced control IgG (Figure 1 and Figure S1). Furthermore, the observed bands under reducing conditions as ~25 and ~50 kilodaltons (kDa), corresponded to the human kappa light chain and human gamma heavy chain, respectively, when analyzed by Western blotting (Figure 2A,B and Figure S2A,B). When probed under non-reducing conditions for the kappa light, a band observed as ~175 kDa, was detected (Figure 2C and Figure S2C), verifying the identity of the properly assembled IgG heterotetramer.

**Figure 3:**
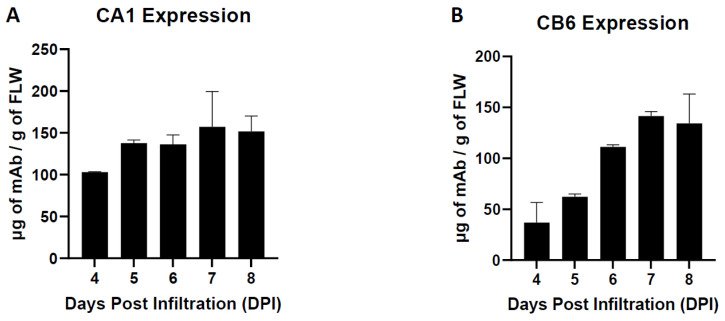
**Temporal expression of CA1 (A) and CB6 (B) in *N. benthamiana*.** Total soluble plant extracts were analyzed over time by sandwich ELISA that detects fully assembled human IgG. Mean ± SEM is plotted from two independent experiments.

The temporal expression of each nAb was examined by a sandwich ELISA that only recognizes the properly assembled heterotetrameric IgG. It was determined that recombinant CA1 and CB6 expression peaked at 157.4 µg/g of fresh leaf weight (FLW) and 141.6 µg/g FLW, respectively, at seven days post agroinfiltration (DPI) (Figure 3A,B). Overall, the high expression of these nAbs in *N. benthamiana*, along with their proper assembly, supported their further development and characterization as SARS-CoV-2 therapeutics.

### 3.2. Plant-Made CA1 and CB6 Recognize SARS-CoV-2 RBD and Neutralize Multiple Variants

**Figure 4:**
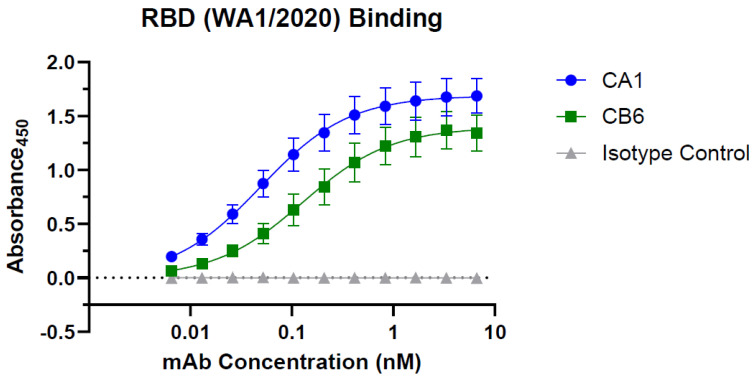
**Antigen-specific recognition of plant-made CA1 and CB6.** Plant-made CA1 and CB6 were serially diluted and incubated with SARS-CoV-2 RBD (WA1/2020) coated on plates. The specific binding of CA1 or CB6 with RBD was detected by an HRP-conjugated secondary antibody. A plant-produced mAb against WNV (E16) was used as the IgG isotype negative control. The absorbance_450_ values presented are mean ± SEM from three independent experiments.

**Figure 5:**
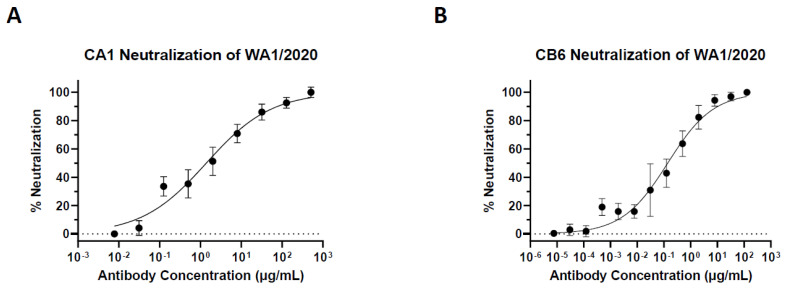
**Neutralization of SARS-CoV-2 by plant-made CA1 and CB6.** Serially diluted plant-made CA1 (**A**) or CB6 (**B**) were mixed with SARS-CoV-2 before adding to Vero E6 cells in a 96-well plate for 24 h. Cells were fixed, permeabilized, and stained for SARS-CoV-2 S protein. Foci were quantified, percent neutralization was calculated, and IC_50_ was determined. Error bars represent SD and at least two independent experiments were performed with technical triplicates.

The plant-made nAbs were tested for specific epitope recognition of the SARS-CoV-2 RBD (WA1/2020 strain). ELISA analysis indicated that both plant-made nAbs have a specific binding activity to their target antigen (Figure 4). Furthermore, the dissociation constants (*K_D_*) for plant-made CA1 and CB6 were calculated to be 0.04938 and 0.1274 nM, respectively, which are similar to what has been reported for the mammalian cell-produced counterparts [15]. With binding activity confirmed, the neutralization potency of the plant-made nAbs was investigated with authentic SARS-CoV-2 in a focus-forming assay (FFA) [46]. Plant-made CA1 and CB6 were found to neutralize the WA1/2020 strain with a half-maximal inhibitory concentration (IC_50_) of 9.29 nM and 0.93 nM, respectively (Figure 5A,B and Table 1). In contrast, an RBD-binding but non-neutralizing mAb (CR3022) produced in plants in parallel did not show any neutralizing activity (Table S1). In addition to the original WA1/2020 strain, CA1 and CB6 were found to also neutralize the more pathogenic B.1.617.2 (delta) variant with an IC_50_ of 89.87 nM and 0.75 nM, respectively (Table 1). We also analyzed the neutralization of plant-made CA1 and CB6 against the mouse-adapted strain of SARS-CoV-2, MA10 [45]. It was discovered that both plant-made antibodies neutralize MA10, with an IC_50_ of 5.15 nM for CA1 and 7.29 nM for CB6 (Table 1), leaving the option open for future examination of their in vivo activity in mouse models.

### 3.3. Plant-Made CB6 Has Synergy with Other Anti-RBD mAbs in Neutralizing SARS-CoV-2

**Figure 6:**
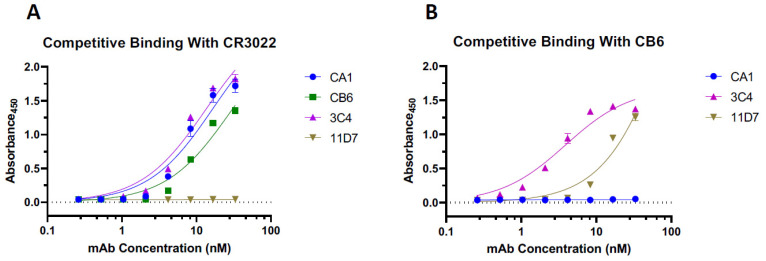
**Competitive Binding to SARS-CoV-2 RBD.** Serial dilutions of each mAb were coated on a 96-well plate prior to incubation with SARS-CoV-2 RBD. Then, either CR3022 (**A**) or CB6 (**B**) conjugated to horseradish peroxidase (HRP) were used to detect either overlapping or complementary binding with each individual mAb. Error bars represent the SD of two independent experiments.

Next, we investigated if the epitopes of plant-made CA1 and CB6 overlap with those of other anti-RBD mAbs that we developed in hybridomas. The results from a competitive ELISA assay indicate that neither CA1 nor CB6 interferes with CR3022 (a non-neutralizing Class 4 SARS-CoV-2 mAb [18]) binding to the RBD (Figure 6A). In addition, we were unable to detect the binding of CB6 to RBD that was already complexed with CA1 (Figure 6B), indicating that CA1 and CB6 compete for RBD binding. Two other anti-RBD mAbs, which we developed in hybridomas, 3C4 and 11D7, were used to help further characterize mAb binding to the RBD. We discovered that 11D7—but not 3C4—has an overlapping epitope with CR3022 (Figure 6A). In contrast, no competitive binding was observed with CB6, in that CB6 was able to bind to the RBD already bound to 3C4 and 11D7 (Figure 6B). This led us to analyze the synergetic potential of CB6 with 3C4 and 11D7 in neutralizing SARS-CoV-2. CB6, 3C4, and 11D7 were diluted to concentrations that are equivalent to the IC_20_, IC_25_, and IC_50_ for each individual mAb. FFA experiments were then performed with diluted CB6, 3C4, and 11D7 either alone, in dual combination with CB6, or as a triple mAb cocktail. Neutralization data obtained from the FFAs were then analyzed using SynergyFinder [47] with both the highest single agent (HSA) [48] and Loewe [49] models to predict synergy at IC_20_, IC_25_, and IC_50_. Analysis of both models indicated that the observed neutralization values of mAb—at all IC concentrations—are higher than the predicted neutralization percentage of the cocktail (Table 2). The predicted neutralization percentage is calculated from the actual neutralization percentage of each individual mAb at a given IC, assuming there is no synergy between the different mAbs in the cocktail. These results indicate that there is neutralization synergy between CB6, 3C4, and 11D7, either as a dual or triple mAb cocktail at all tested concentrations (Table 2). For example, we observed synergy from the CB6 + 3C4 combination at IC_50_ concentrations with an increase in the neutralization of 9.88% and 5.59% using the HSA and Loewe models, respectively (Table 2). At IC_50_ concentrations, the CB6 + 11D7 combination saw an increase of 29.83% (HSA model) and 29.78% (Loewe model) (Table 2), while the 3 mAb combination resulted in increases of 41.01% and 22.9% for the HSA and Loewe models, respectively (Table 2).

## 4. Discussion

The ongoing pandemic caused by SARS-CoV-2, and the emergence of viral variants, urges the continual development of therapeutic strategies to counteract viral infection. Here, both CA1 and CB6 (also known as etesevimab, JS106, or LY-CoV016) reached high levels of accumulation in *N. benthamiana* within one week of gene delivery. These expression levels are higher than that of plant-made anti-SARS-CoV-2 S mAbs reported to date [34,53]. CA1 and CB6 were also purified to high homogeneity, like other plant-produced mAbs [41,43,53,54]. Specifically, the major SDS-PAGE bands from protein A-purified mAbs from plants were identified as mAb components, with the purity comparable to a one-step-purified, mammalian-made mAb. Furthermore, the plant-made nAbs retained recognition for the WA1/2020 strain RBD with dissociation constants of 0.04938 nM for CA1 and 0.1274 nM for CB6. These are in a similar range to the reported *K_D_* for the mammalian-made counterparts [15].

Our plant-made CA1 and CB6 preserved their parental neutralizing capacity against the WA1/2020 with an IC_50_ of 9.29 nM and 0.93 nM, respectively, which is in line with the published neutralization data on authentic SARS-CoV-2 [15]. Notably, CR3022, a mAb produced in *N. benthamiana* plants in parallel with CA1 and CB6, did not show any neutralizing activity. This indicates that the observed neutralizing potency of CA1 and CB6 is not caused by the interference of any potential residual host protein or plant impurity. Furthermore, we report that these two plant-made nAbs also neutralize the delta variant with an IC_50_ of 89.87 nM for CA1 and an IC_50_ of 0.75 nM for CB6. The approximately one order of magnitude decrease in the neutralizing potency of plant-made CA1, however, indicates that this nAb has lost practical utility against this variant and will likely have similarly decreased neutralizing capacity against emerging variants derived from the delta lineage. In contrast, plant-made CB6 maintained a neutralizing ability against the delta variant, suggesting that it has more potential in being an effective therapeutic against emerging variants of SARS-CoV-2. CA1 and CB6 also neutralized a mouse-adapted strain of SARS-CoV-2, termed MA10 [45], with an IC_50_ of 5.15 nM and 7.29 nM, respectively. It is encouraging that both the nAbs we tested resulted in minimal changes of IC_50_ against MA10, as this sets the foundation for future animal studies utilizing these plant-made nAbs.

To assess candidates for synergetic use in a nAb cocktail, we utilized different variations of competitive ELISAs. CR3022 is a well-characterized antibody derived from a convalescent SARS-CoV patient [55]. It cross-reacts with the SARS-CoV-2 RBD but does not overlap with the ACE2 binding site and does not neutralize SARS-CoV-2 [19,34,56]. Our data show that plant-made CA1 and CB6 do not have overlapping epitopes with CR3022 but do with each other. This was not unexpected, as it had been previously observed that CA1 and CB6 compete for binding to the SARS-CoV-2 RBD [15], making them undesirable cocktail partners. Moreover, based on the epitope classification system [19,20], CR3022 is a Class 4 mAb, CB6 is a Class 1 mAb, and CA1 remains an unclassified mAb due to a lack of structural data from the CA1-RBD complex. Based on the data from our study, we can speculate that CA1 is likely to be a Class 1 or 2 mAb since its epitope overlaps with CB6 [19]. Regardless, the neutralization data support further development of the more potent and variant-neutralizing plant-made CB6 as a therapeutic nAb over plant-made CA1. To identify mAbs that have non-overlapping RBD binding sites and, thereby, may have neutralization synergy with one of the plant-made mAbs in a cocktail, we developed additional mAbs by hybridoma generation in mice. Two hybridoma-derived nAbs, 3C4 and 11D7, were found to have non-overlapping epitopes with the plant-made nAbs. This provided a foundation on which to build a novel antibody cocktail and highlighted the role of a plant-made nAb in the synergistic cocktail. Although 11D7 has an overlapping epitope with the non-neutralizing CR3022, neither of the hybridoma-made nAbs had overlapping epitopes with plant-made CB6, indicating they would be ideal candidates for testing neutralizing synergy with plant-made CB6.

This is the first assessment and report to describe the potential utility of a plant-made, SARS-CoV-2 nAb in an antibody cocktail. Cocktails of nAbs are promising therapies for the treatment of SARS-CoV-2 and several have been granted EUA by the FDA, and more are still in development [23,57,58]. Given the revision of EUAs for some mAb-based therapies, due to reduced activity against the omicron variant [59,60,61], the development of nAb cocktails utilizing more than two nAbs with non-overlapping epitopes may be the best approach to overcoming viral immune evasion in the future. The synergy analysis in this study highlights a potential new cocktail incorporating plant-made CB6 (etesevimab), along with our hybridoma-made 3C4 and 11D7. All combinations with either a two- or three-nAb cocktail resulted in increased neutralizing activity, indicating synergy. Specifically, the CB6 + 11D7 combination resulted in almost a 30% (29.83% and 29.78%, using both models) increase in neutralizing potency when using each nAb at their respective IC_50_. This observation was nearly identical in the three nAb combinations, suggesting that 3C4, when used with CB6 and 11D7, contributes minimal neutralizing synergy when used in this triple combination. However, studies are ongoing to assess and further characterize the synergetic nature of 3C4 with 11D7 to determine if the synergy we observed with plant-made CB6 would produce a useful, triple nAb cocktail that minimizes escape mutants of SARS-CoV-2.

In recent years, progress in the field of plant-made biologics has offered the opportunity for plant-derived mAbs to become more realistic contenders in human therapeutics [26]. One of the major concerns for the earlier plant-based expression systems was that they produce mAbs with plant-specific glycans that are different from those produced in mammalian cells. However, this challenge has been overcome by glycoengineering of host plants by deleting or suppressing the expression of plant-specific glycan genes and/or inserting mammalian glycosylation genes [28]. Studies have shown that mAbs produced in glycoengineered plants do not carry any plant-specific glycans, eliminating the concern for immunogenicity and the potential risk of adverse effects from plant-produced mAbs [62,63]. Furthermore, mAbs made in glycoengineered plants usually carry a homogenous N-glycan structure compared to the mixture of multiple glycans exhibited by the same mAbs produced in CHO cells [42,64]. Therefore, mAbs produced in glycoengineered plants not only forgo the issue of non-native glycans but may outperform their CHO-derived counterparts due to their defined and uniform carbohydrate moieties. CB6 and CA1 were produced with the stable *N. benthamiana* plant line ΔXF, where endogenous plant β1,2-xylosyltransferase and α1,3-fucosyltransferase genes were down-regulated by RNAi [63]. This plant line has been shown in multiple studies to generate mAbs with >90% homogeneous glycans of the GnGn structure and without plant-specific glycans and, in parallel, provide a more consistent human N-glycan than CHO-derived mAbs [64,65]. Therefore, CB6 is expected to carry the human GnGn glycoform and have potential utility in cocktails for human application.

As a platform, plants have also demonstrated the capability of producing mAbs with the quality and characteristics that match those produced in CHO cells [26]. For example, a plant-made anti-HIV mAb (2G12) has been extensively characterized in a clinical study and was found to meet all regulatory specifications for human application [66]. Still, other challenges persist, including the lack of interest by large pharmaceutical companies. The absence of a clear regulatory pathway in the past is partially responsible for the inertia of large pharmaceutical companies, as regulatory agencies such as the FDA were uncertain about how to fit biologics made by plants into their regulatory approval framework, established mostly for CHO cell-derived biologics. However, the approval of ELELYSO by the FDA, along with the clinical development of ZMapp and 2G12, has cleared the regulatory pathway and slowly warmed up the interest of large pharmaceutical companies toward plant-made mAbs [66,67,68,69]. For example, Pfizer has entered into an agreement to license the worldwide rights for commercializing ELELYSO, and the very recent approval of a plant-made COVID-19 vaccine co-developed by Medicago and GlaxoSmithKline (GSK) [70] should facilitate the commercial development of plant-made mAbs and streamline the approval of those that have shown safety in human clinical trials.

To advance our plant-made mAbs into future in vivo studies in animal models, further extensive physicochemical and structural characterization of their identity and purity, including post-translational modifications, fragmentation, and aggregation, and plant-derived impurities, will be performed to ensure their expected quality. Additional chromatographic steps will also be included in downstream processing to further purify the plant-derived mAbs to ensure that mAb fragments, aggregates, contaminants, and impurities, including plant-derived impurities and host proteins, are efficiently eliminated prior to in vivo studies, as indicated by previous studies [43,66]. 

## 5. Conclusions

This study demonstrates the robustness and versatility of plant-based systems in rapidly developing neutralizing mAbs and evaluating their functional synergy in controlling infectious diseases. Overall, this study supports the further development of plant-based mAb therapeutics that have utility in the ongoing SARS-CoV-2 pandemic.

## Figures and Tables

**Table 1:** Half-maximal inhibitory concentrations (IC_50_) of plant-made CA1 and CB6 against SARS-CoV-2 strains.

Strain (Variant)	CA1 (IC_50_)	CB6 (IC_50_)
WA1/2020	9.29 nM	0.93 nM
B.1.617.2 (Delta)	89.87 nM	0.75 nM
Mouse-Adapted (MA10)	5.15 nM	7.29 nM

**Table 2:** **Neutralization synergy analysis of plant-made CB6 with other anti-SARS-CoV-2 RBD neutralizing mAbs.** FFA experiments were performed for each individual nAb as well as nAb combinations with each nAb at concentrations that correspond to its IC_20_, IC_25_, and IC_50_. Neutralization data from two independent experiments were analyzed using the HSA and Loewe models (SynergyFinder.org). Predicted neutralization values represent the percent neutralization of nAb combinations, where there is no synergistic interaction between different nAbs in the cocktail at the indicated IC value. These predicated neutralization values were calculated from the actual neutralization data of each individual nAb in the cocktail, assuming there is no interaction between the nAbs in the cocktail.

Cocktail Combination & Concentration	Observed Percent Neutralization	HSA Predicted Neutralization	Loewe Predicted Neutralization
CB6 + 3C4 (IC_20_)	51.66%	33.97%	33.94%
CB6 + 11D7 (IC_20_)	63.18%	33.97%	36.06%
CB6 + 3C4 +11D7 (IC_20_)	60.84%	33.7%	36.08%
CB6 + 3C4 (IC_25_)	58.65%	33.15%	44.54%
CB6 + 11D7 (IC_25_)	78.2%	33.15%	42.42%
CB6 + 3C4 + 11D7 (IC_25_)	77.87%	34.1%	41.31%
CB6 + 3C4 (IC_50_)	67.09%	57.21%	61.5%
CB6 + 11D7 (IC_50_)	87.04%	57.21%	57.26%
CB6 + 3C4 + 11D7 (IC_50_)	88.49%	47.48%	65.59%

## Data Availability

The data presented in this study are contained within this article.

## Supplementary Materials

The following supporting information can be downloaded at: https://www.mdpi.com/article/10.3390/vaccines10050772/s1, Figure S1: Purification of CB6 from *Nicotiana benthamiana* leaves. CB6 purified by Protein A affinity chromatography was subjected to electrophoresis on a 4–20% gradient polyacrylamide gel under reducing (Lane 1) or non-reducing (Lane 3) conditions. Total protein content was stained with Coomassie Brilliant Blue. Approximately 2.5 µg of IgG was loaded in each lane. Lanes 1 and 3, plant-made CB6; Lanes 2 and 4, a mammalian cell-produced anti-West Nile virus E protein (E16) IgG control; M, molecular weight ladder. One representative gel of several experiments is shown. Figure S2: Western blot analysis of plant-made CB6. Plant-made CB6 was subjected to SDS-PAGE under reducing conditions (A,B) and non-reducing conditions (C). Proteins were transferred to a PVDF membrane after separation and a horseradish peroxidase-conjugated goat anti-human kappa (A,C) or goat anti-human IgG (B) antibody was used to detect the light chain and heavy chain, respectively. Lane 1, plant-made CB6; Lane 2, mammalian cell-produced anti-West Nile virus E protein (E16) IgG. Shown are representatives of multiple independent experiments. Table S1: Half-maximal inhibitory concentrations (IC_50_) of plant-made CR3022 against SARS-CoV-2.
