# Supplementary material for: Potential for a Plant-Made SARS-CoV-2 Neutralizing Monoclonal Antibody as a Synergetic Cocktail Component
Source: Vaccines (Basel). 2022 May 12;10(5):772. doi: 10.3390/vaccines10050772 (PMC9145534; doi:10.3390/vaccines10050772)
Supplement: Supplementary file 1 [file vaccines-10-00772-s001.zip › vaccines-1707444-supplementary.pdf]

Supplementary Materials:

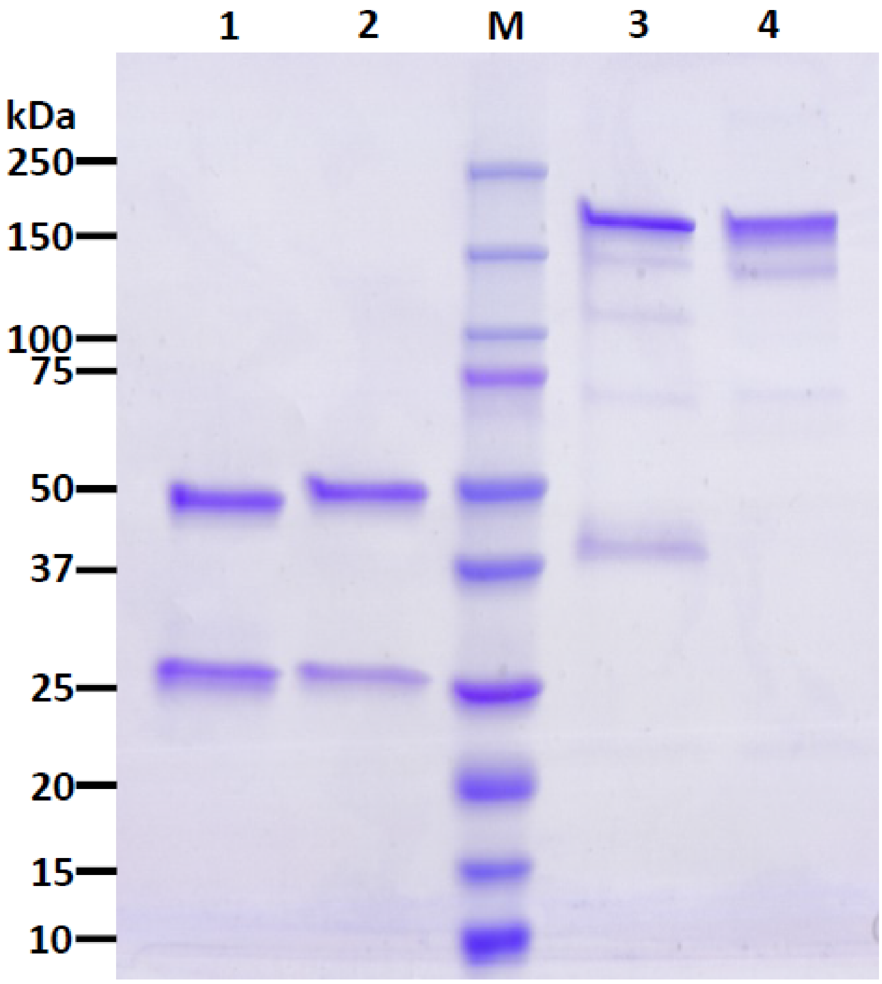

Figure S1. Purification of CB6 from *Nicotiana benthamiana* leaves. CB6 purified by Protein A affinity chromatography was subjected to electrophoresis on a 4–20% gradient polyacrylamide gel under reducing (Lane 1) or non-reducing (Lane 3) conditions. Total protein content was stained with Coomassie Brilliant Blue. Approximately 2.5 μg of IgG was loaded in each lane. Lanes 1 and 3, plant-made CB6; Lanes 2 and 4, a mammalian cell-produced anti-West Nile virus E protein (E16) IgG control; M, molecular weight ladder. One representative gel of several experiments is shown.

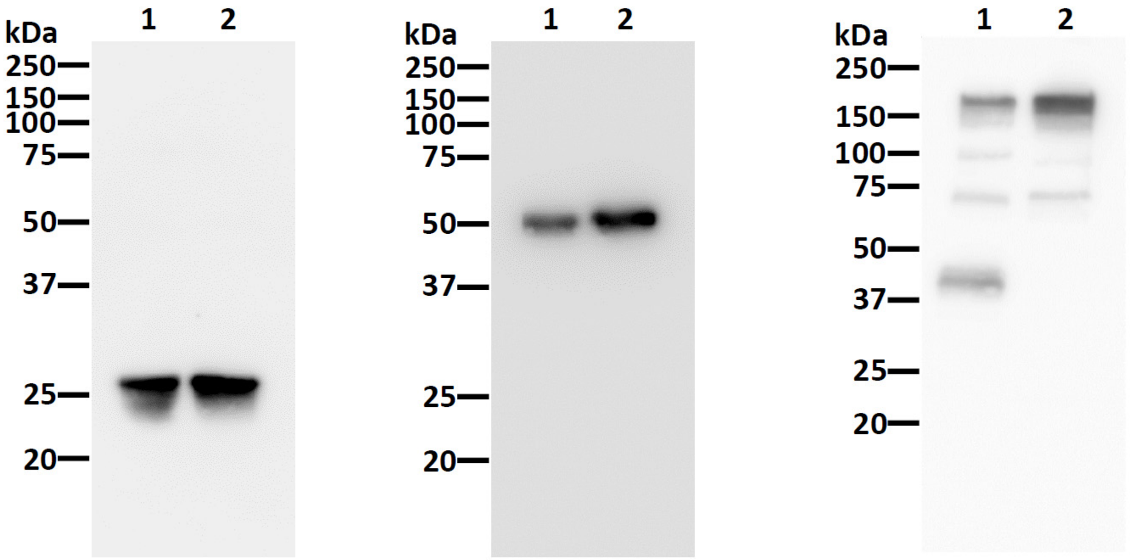

Figure S2. Western blot analysis of plant-made CB6. Plant-made CB6 was subjected to SDS-PAGE under reducing conditions (A,B) and non-reducing conditions (C). Proteins were transferred to a PVDF membrane after separation and a horseradish peroxidase-conjugated goat anti-human kappa (A,C) or goat anti-human IgG (B) antibody was used to detect the light chain and heavy chain, respectively. Lane 1, plant-made CB6; Lane 2, mammalian cell-produced anti-West Nile virus E protein (E16) IgG. Shown are representatives of multiple independent experiments.

Table S1. Half-maximal inhibitory concentrations (IC_{50}) of plant-made CR3022 against SARS-CoV-2.

Strain (Variant)	CR3022 (IC50)
WA1/2020	Not detectable
